# IL-1β and HMGB1 are anti-neurogenic to endogenous neural stem cells in the sclerotic epileptic human hippocampus

**DOI:** 10.1186/s12974-021-02265-1

**Published:** 2021-09-21

**Authors:** Malik Zaben, Niels Haan, Feras Sharouf, Aminul Ahmed, Lars E. Sundstrom, William P. Gray

**Affiliations:** 1 Brain Repair and Intracranial Neurotherapeutics (BRAIN), Biomedical Research Unit, Division of Psychological Medicine and Clinical Neurosciences, School of Medicine, Cardiff University, Cardiff, CF24 4HQ UK; 2 Institute of Psychological Medicine and Clinical Neurosciences, National Institute for Neuroscience and Mental Health Research, Cardiff University, Cardiff, UK; 3 Clinical Neurosciences, Faculty of Medicine, University of Southampton, Southampton, UK; 4 Sundstrom Innovation Limited, 14 Marine Parade, Clevedon, BS21 7QS UK

**Keywords:** Neurogenesis, Epilepsy, Neuroinflammation, IL-1β and HMGB1

## Abstract

**Background:**

The dentate gyrus exhibits life-long neurogenesis of granule-cell neurons, supporting hippocampal dependent learning and memory. Both temporal lobe epilepsy patients and animal models frequently have hippocampal-dependent learning and memory difficulties and show evidence of reduced neurogenesis. Animal and human temporal lobe epilepsy studies have also shown strong innate immune system activation, which in animal models reduces hippocampal neurogenesis. We sought to determine if and how neuroinflammation signals reduced neurogenesis in the epileptic human hippocampus and its potential reversibility.

**Methods:**

We isolated endogenous neural stem cells from surgically resected hippocampal tissue in 15 patients with unilateral hippocampal sclerosis. We examined resultant neurogenesis after growing them either as neurospheres in an ideal environment, in 3D cultures which preserved the inflammatory microenvironment and/or in 2D cultures which mimicked it.

**Results:**

3D human hippocampal cultures largely replicated the cellular composition and inflammatory environment of the epileptic hippocampus. The microenvironment of sclerotic human epileptic hippocampal tissue is strongly anti-neurogenic, with sustained release of the proinflammatory proteins HMGB1 and IL-1β. IL-1β and HMGB1 significantly reduce human hippocampal neurogenesis and blockade of their IL-1R and TLR 2/4 receptors by IL1Ra and Box-A respectively, significantly restores neurogenesis in 2D and 3D culture.

**Conclusion:**

Our results demonstrate a HMGB1 and IL-1β-mediated environmental anti-neurogenic effect in human TLE, identifying both the IL-1R and TLR 2/4 receptors as potential drug targets for restoring human hippocampal neurogenesis in temporal lobe epilepsy.

## Background

Neurogenesis is a restricted event in the adult human brain largely confined to the dentate gyri [1] where it supports certain forms of hippocampal dependent learning and memory [2–4]. It plays a role in both the acquisition [5, 6] and retrieval [5] of spatial relational memory, and appears to be particularly important for the fine pattern separation necessary for hippocampal dependent allocentric spatial learning [6], as well as regulating forgetting [7]. Status epilepticus permanently alters hippocampal neurogenesis [8–10] in most models of status-induced epilepsy, such that it is chronically impaired both in level [11] and connectivity [12, 13]. Isolated neural stem cell culture studies on resected tissue from mesial temporal lobe epilepsy (mTLE) patients have confirmed this neurogenic impairment, which is associated with impaired learning and memory performance [4]. Thus, impaired neurogenesis may be one possible mechanism for the learning and memory deficits seen in patients with mTLE.

We have shown that restoring neurogenesis to normal levels in chronically epileptic animals using the antidepressant fluoxetine completely corrects spatial learning impairment [14], demonstrating that reduced neurogenesis is pharmacologically reversible, and suggesting that altered neurogenesis is a key mechanism underlying spatial learning impairment in mesial temporal lobe epilepsy. However, the underlying mechanisms of the initiation, maintenance and potential reversibility of altered neurogenesis in chronic human temporal lobe epilepsy remain unknown.

Immune cells [15] and cytokines [16] are significant modulators of neurogenesis under normal and pathological conditions involving acute and chronic inflammation [17]. Interleukin 1 β (IL-1β) and the high mobility group box 1 (HMGB1) protein are key initiators of neuroinflammation [18] instigating both acute seizure mechanisms and epileptogenesis [19–21]. Increased IL-1β and HMGB1 levels have been found in human brain tissue resected at surgery for medically refractory mesial temporal lobe epilepsy and focal cortical dysplasias [21, 22] but active translocation has not been demonstrated in epileptic human tissue. Studies of rodent hippocampal neurogenesis have demonstrated potent anti-neurogenic effects of IL-1ß mediated via the interleukin-1 receptor (IL-1R) [23, 24]. HMGB1 has both pro [25] and anti-neurogenic effects [26–28] across different disease models, the interpretation of which is complicated by the indirect effects of co-activated proinflammatory pathways.

Although Coras et al. [4] showed a reduced proliferative capacity of hippocampal stem cells in culture from patients with low cognitive performances, the mechanism of such a reduction and therefore its reversibility remains unknown. Its elucidation however, is critical for the development of rational targeted pharmacological therapies to restore cognition in these patients; an unmet need identified as central to improving the health of patients with temporal lobe epilepsy [29, 30].

Given the close association of HMGB1 and IL-1β in the initiation, propagation and maintenance of seizure activity as well as epileptogenesis, the current study was undertaken to investigate the hypothesis that the pro-inflammatory sclerotic hippocampal microenvironment of adult patients with chronic mTLE has an anti-neurogenic effect that is at least partly mediated by HMGB1 and/or IL-1β signalling and if so, to examine whether this effect can be pharmacologically reversed.

## Methods

### Tissue preparation

We used excess primary human cortical and hippocampal excised at selective hippocampectomy epilepsy surgery (National Health Service ethics approvals: Southampton and Southwest Hampshire LRECb No 07/H0504/195; Wales Neuroscience Research Tissue Bank (19/WA/0058). Cortical and hippocampal (with histologically confirmed sclerosis) tissues were micro-surgically dissected through a trans-sylvian approach (Fig. 2A) from mTLE patients with intractable seizures and poor memory (Table 1). Cells from the same patient were either grown as cell aggregates on air-liquid interface maintaining the cell components of the parent tissue microenvironment (3D cultures), as floating neurospheres in an ideal environment with growth factors, or as two-dimensional (2D) monolayers under standard growth conditions (see below).

**Table 1:** The clinical characteristics of the patients from whom the tissue was collected

#	Patient ID (Date of surgery)	Sex	Age at the time of surgery	Duration of epilepsy	Side of resection	Histology findings	Seizure type	Type of memory/learning deficit (if present)
1	24.05.11	M	59	48 years	R	Hippocampal Sclerosis	Partial, leading to TC	Non-verbal
2	06.07.10	M	27	20 years	L	Hippocampal Sclerosis	Complex partial GTCS	Non verbal
3	29.09.09	F	59	41	R	Hippocampal Sclerosis	Complex partial	Non-verbal and Low cognition
4	22.01.09	M	52	25	R	Hippocampal Sclerosis	Complex partial	Non-verbal
5	12.11.08	F	44	6	R	Hippocampal Sclerosis	Complex partial, secondary generalised	Visuo-spatial
6	03.09.08	M	30	21	R	Hippocampal Sclerosis	Complex Partial	Verbal
7	28.10.2013	F	54	50 years	L	Hippocampal sclerosis	Focal symptomatic	Verbal
8	25.11.13	F	25	10 years	R	Hippocampal Sclerosis	Focal symptomatic	Verbal
9	23.06.2014	F	48	9 years	L	Hippocampal sclerosis	Focal symptomatic	Verbal
10	22.09.2014	F	54	44 years	L	Hippocampal Sclerosis	Focal symptomatic	Verbal
11	10.11.2014	M	46	30 years	L	Hippocampal Sclerosis	Complex partial GTCS	Verbal
12	17.11.2014	F	27	14 years	R	Hippocampal Sclerosis	Complex partial	Non-verbal and Low cognition
13	02.02.2015	F	60	15 years	L	Hippocampal Sclerosis	Focal symptomatic	Border line verbal and non-verbal impairment
14	20.07.2015	M	34	12 years	R	Hippocampal Sclerosis	Complex partial	Verbal and non-verbal
15	01.09.2015	F	24	12 years	L	Hippocampal Sclerosis	Complex partial	Verbal

**Fig. 1:**
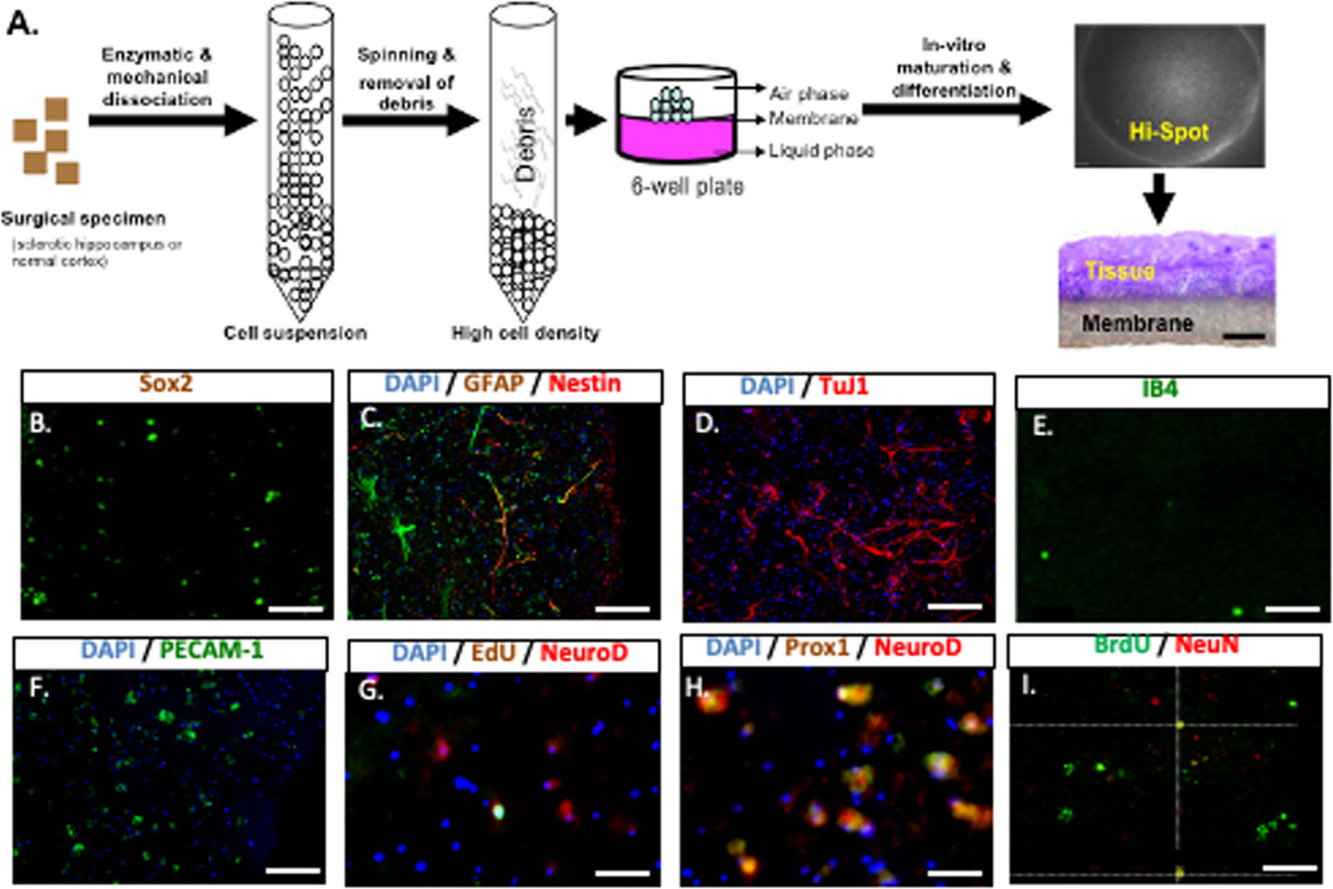
3D cultures generated from sclerotic hippocampi of patients with mTLE replicate components of the in-vivo stem cell niche. **A** Schematic representation of air-liquid interface 3D culture generation. Tissue sections for immunohistochemical staining were obtained by cryosectioning. **B** Early neuronal progenitors (Sox2^+^) are present in our 3D cultures (Scale bar 100 μm). **C** Both nestin^+^ GFAP^+^ type 1 cells and nestin^+^ GFAP^−^ type 2a cells, as well as nestin^−^ GFAP^+^ astrocytes are present (scale bar 100μm). **D** The 3D cultures also contain many immature (NeuroD1^+^) neurons, some of which have been formed in culture, as indicated by EdU incorporation (Scale bar 100 μm). **E** The NeuroD1^+^ neurons are dentate gyrus derived, as shown by expression of the developmentally specific granule cell neuron marker Prox1 (scale bar 100 μm). **F** βIII tubulin staining reveals the complex morphology of neurons in 3D culture (Scale bar 100 μm). **G** The presence of recently generated mature post-mitotic neurons is indicated by NeuN and BrdU co-immunoreactivity, indicating generation in culture (scale bar 25 μm). **H** Small numbers of IB4^+^ microglia are present (scale bar 25 μm). **I** Many of the remaining cells are PECAM^+^ endothelial cells. scale bar 10μ (scale bar 100 μm)

Epileptic hippocampal and patient-matched control cortical surgical samples of 0.5–2 cm^3^ were transferred in Gey’s buffer (Sigma-Aldrich), meninges and blood vessels removed and mechanically chopped (McIlwain Tissue Chopper). Tissue was incubated in Dulbecco’s modified Eagle medium (DMEM) containing 1 μM MK-801 (Sigma-Aldrich) for 5 min and 2 mg/ml Papain (22.0 U/mg, Sigma-Aldrich) for 1 h at 37 °C, triturated and passed through a 70 μm cell strainer (BD, Biosciences). The cell-containing fraction was collected after centrifugation (2000 rpm) (Fig. 1A) and diluted into cortical cell culture medium (DMEM with 4.5 mg/ml glucose (GIBCO®), 2% B27 (Life Technologies), 2 mM glutamine (Invitrogen), 20% foetal bovine serum (FBS) (GIBCO®), 5% horse serum (GIBCO®), 10% Ham’s F-12 (3:1) (GIBCO®), 10 nM HEPES (GIBCO®), 1% antibiotics (Invitrogen)). Cell viability was determined using trypan blue exclusion (Sigma-Aldrich), and quantified using a haemocytometer.

#### 3D cell culture generation

We developed a novel 3D human primary culture system based on a technique using rodent tissue [31] in an attempt to model the environmental effects on neurogenesis in human mTLE. To generate these cultures, viable cells at a high density (50,000 cell/μl) were plated in 5 μl drop of cell suspension placed onto air-liquid interface onto 6 mm polytetrafluoroethylene membrane discs placed onto the membranes of Millicell CM inserts in 6-well plates (3 discs/well) with cortical medium and incubated at 37 °C in humidified air with 5% CO_2_, with 3-day medium changes (Fig. 1A). Cultures were maintained in cortical medium with medium change twice weekly.

#### Neurosphere assay

For neurosphere cultures, 10 ml of cell suspension from the same patient was plated at a density of 100,000 cells/ml into T75 culture flasks in neurosphere cell culture medium (Neurobasal A (Invitrogen), 2% B27 (Life Technologies), 2 mM Glutamine (Invitrogen), 2 μg/ml Heparin (Sigma-Aldrich), 1% antibiotics (Invitrogen), 20 ng/ml Fibroblast growth factor (FGF-2) (Roche) and 20 ng/ml epidermal growth factor (EGF) (Chemicon)); under the same culture and incubation conditions of 3D cell cultures (see above). At 14 Days in vitro DIV, clusters of proliferating cells were observed. Established neurospheres were not seen until after 30 DIV (primary neurospheres). These were then passaged, dissociated and expanded to generate secondary neurospheres. Neurogenesis was then quantified by dissociation of secondary neurospheres (at least 100 μm in diameter) into a single cell suspension and plated as adherent monolayer cultures in growth factor-free medium for 10 DIV before immunohistochemical staining for βIII tubulin (TuJ1) expression was carried out.

#### Monolayer cell cultures

In a reductionist model, cells from the same patient were also processed to generate 2D monolayer cell culture. In this set of experiments, cells were grown on standard poly-l-lysine, poly-l-ornithine and laminin coated glass coverslips at a density of 1 × 10^6^/ml standard culture medium ((Neurobasal A (Invitrogen), 2% B27 (Life Technologies), 2 mM Glutamine (Invitrogen), 2 μg/ml Heparin (Sigma-Aldrich), 1% Antibiotics (Invitrogen)) in same culture and incubation conditions outlined for 3D cultures. Cell culture medium was changed every 3 days.

#### Drugs and treatment

To block IL-1β, 2D or 3D cultures were treated with 100 ng/ml IL-1Ra at 2-h post plating for the duration of the experiment. Similarly, BoxA was added to cultures at 2 h after platting at a concentration of 100 ng/ml. Monolayers cultures were treated with 10 ng/ml of IL-1β at 2 h post plating and for the duration of the experiment.

### Quantification of neurogenesis

**Fig. 2:**
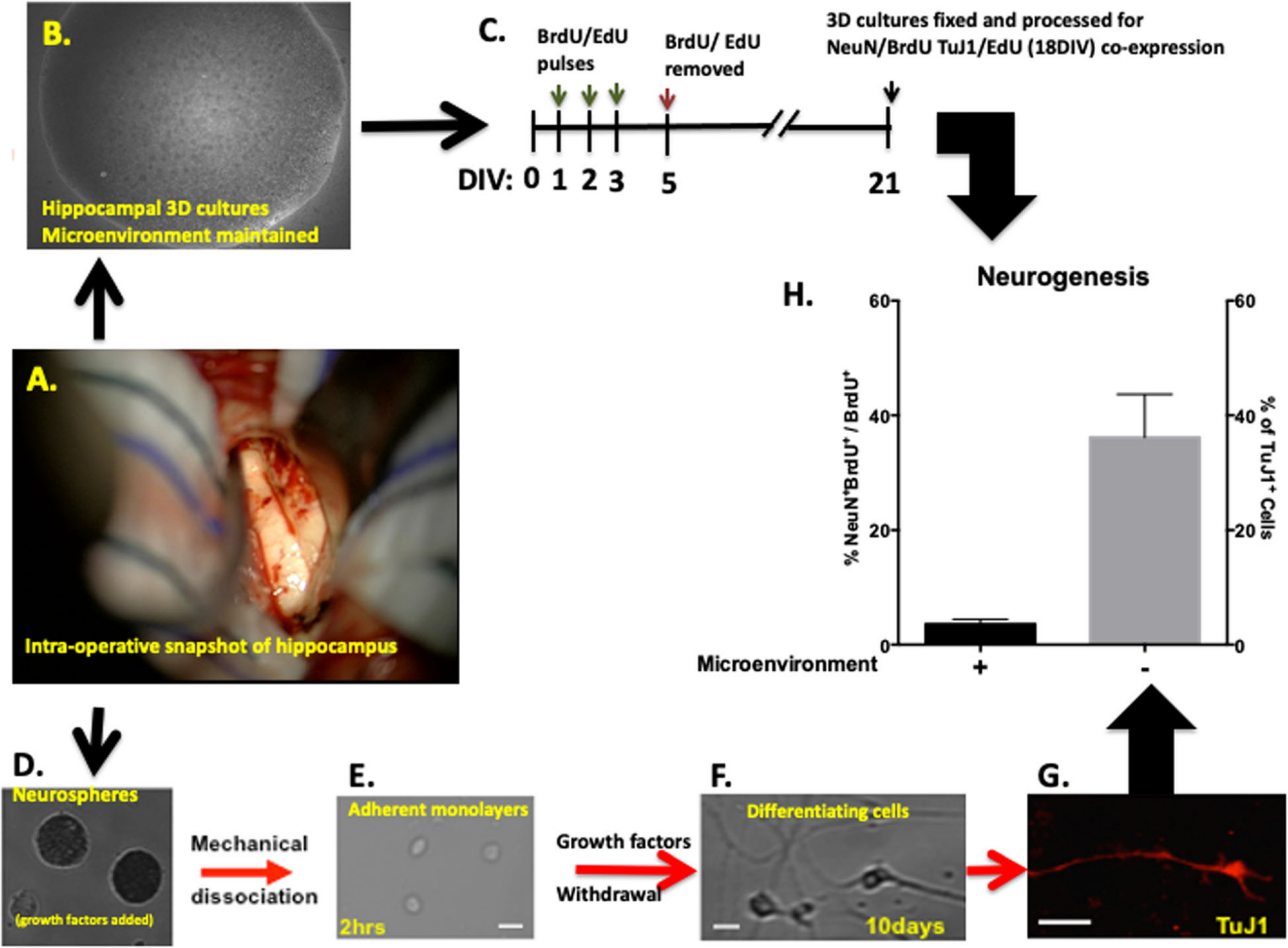
The microenvironment of parent hippocampal sclerotic tissue is anti-neurogenic. **A** An intraoperative photograph of the human hippocampus at the end of a trans-sylvian dissection from a patient with mTLE. **B** Bright-phase snapshot (5× field) of 3D hippocampal slice culture at 21DIV. **C** A schematic outline of the pulse and chase protocol used to quantify neurogenesis in 3D cultures using a BrdU or EdU and NeuN or TuJ1, respectively. **D**–**G** Quantification of neurogenesis (TuJ1^+^cells) in cells dissociated from secondary neurospheres generated from the same patient. **H** The neurogenic capacity of cells grown in ideal microenvironment was ten times higher than those grown in 3D hippocampal cultures generated from the same patient Values are means ± SE based on samples generated from 3 different patients (scale bar for neurosphere cultures is 100 μm and for monolayer adherent cultures is 10 μm)

Neurogenesis was quantified in 3D cultures using a Bromodeoxyuridine/5-bromo-2′-deoxyuridine (BrdU)/NeuN or EdU/Class III β-tubulin (TuJ1) pulse and chase protocols. Briefly, 3D cultures under different conditions were incubated with 20 μM BrdU or EdU immediately after seeding. Three further pulses were given on day 1, 2 and 3 before cultures were washed thoroughly free of BrdU or EdU at 5DIV (Fig. 2C). Cultures were then grown for 14 or 21 DIV before being fixed and immuno-processed for EdU/TuJ1 or BrdU/NeuN expression; respectively. Neurogenic capacity was measured as the percentage of BrdU/NeuN or EdU/TuJ1 co-expressing cells over total BrdU or EdU positive cells, respectively (Fig. 2C).

Neurospheres’ neurogenic capacity was assessed after secondary neurospheres were dissociated into a single cell suspension (100,000 cell/ml) and grown as adherent monolayer cultures in the absence of growth factors (to allow differentiation) before cells were fixed and processed for the expression of the neuronal marker (TuJ1^+^) and counterstained with 4′,6-diamidino-2-phenylindole (DAPI). The percentage of neuronal (TuJ1^+^) cells with respect to total number of cells was then determined under different conditions (Fig. 2D–G).

#### LDH assay

Cell viability was assessed in culture supernatants at time of cell culture medium change. Aliquots of cell culture supernatant at each time point were analysed using the Pierce LDH cytotoxicity assay kit (Thermo Fisher Scientific, 88954) according to the kit protocol. Briefly, 50 μl of each sample was added to triplicate wells in a 96-well plate, along with the kit positive control and samples of media which had not been exposed to cells. Then, 50 μl of the kit reaction mix was added to each well and the plate was incubated at room temperature, protected from light, for 30 min. Further, 50 μl of stop solution was then added to each well. Using a plate reader and associated software (BMG Labtech, CLARIOstar), the absorbance for each well was measured at 490 nm and 680 nm. The absorbance at 680 nm was subtracted from the absorbance at 490 nm for each well. This was then normalised to the mean measurement from the appropriate blank media, and then the mean value was calculated for the triplicate wells. Data were plotted using GraphPad Prism.

#### ELISA assay

The levels of IL-1β and HMGB1 were measured in cultures’ supernatants at the indicated time points using enzyme-linked immunosorbent assay (ELISA) kits (R & D systems and IBL International, respectively) according to the manufacturer’s protocol. After preparing the calibrators, quality controls and samples, plates were loaded and a reproducible standard curve generated. Then, 100 μl of human IL-1β or HMGB1 conjugate was added to each well and the plate incubated for 2 h at room temperature. Optical density was determined on a microplate reader set to 450 nm. The absorbance of the resulting colour change was measured spectrophotometrically and calculated as proportional to the HMGB1 or IL-1β calibrators’ concentrations.

#### PCR assay

Total RNA was extracted from cultured hippocampal and cortical 3D cell cultures using trizol (Sigma-Aldrich) and directly reverse-transcribed to complementary DNA (cDNA) using SuperScript™ III Cells Direct cDNA Synthesis Kit (Invitrogen™ life technologies). The cDNA was then amplified by one-step polymerase chain reaction (PCR) kit (human Custom real-time PCR assay for use with SYBRgreen chemistry) (PrimerDesign Ltd, Southampton) in a real-time thermocycler (Rotor-Gene 6000, Corbett Robotics. Ltd). IL-1R primers were: *Forward*: ATAGTCATTATCTGCTCTGCTACC and *Reverse*: TGGCAAGAGGTCAACGATTAC (PrimerDesign Ltd, Southampton). β-actin was used as a house keeping gene (Accession:NM_016232, Anchor:922, Amplicon length: 125) (PrimerDesign Ltd, Southampton). After results were normalised against the housekeeping gene β-actin, raw data was processed and analysed using the Comparative Ct method (2[-Delta Delta C(t)] method (2[-ΔΔCt]) [32].

#### Western blot assay

3D cortical or hippocampal cultures were lysed in CellLyticMT lysis buffer (Sigma-Aldrich) with a protease inhibitor mix (Roche) for 15 min on ice. Lysates were spun at 15,000 *g* for 20 min at − 4 °c. A standard Bradford assay (Bio-Rad) was performed. The appropriate volume of 5× Lamelli buffer was added to the supernatant. Samples were heated at 75 °C for 10 min. Eighty micrograms of protein was loaded onto a 10% SDS-polyacrylamide gel and run at 100 V constant voltage. The gel was blotted onto a nitrocellulose membrane for 2 h at 25 mV constant current. For development, the membrane was blocked with 2% ECL advanced blocking solution in Tris-buffered saline–Tween (0.1%) for 2 h. The supernatant protein concentration was determined using a BCA quantification kit (Thermo Fisher). Primary antibodies (rabbit anti-Class III β-tubulin diluted 1:1000 (Millipore), mouse anti-NeuroD1 diluted 1:1000 (Abcam), 1:150 goat anti-IL1R (Santa Cruz) and rabbit anti-GAPDH diluted 1:1000 (Cell Signalling) were applied overnight at 4 °C and then detected using species appropriate HRP conjugated secondary antibodies diluted at 1:5000 (Jackson Immunoresearch), The membrane was developed using the ECL Advanced Western Blotting Detection kit (Amersham Biosciences.) and visualised using ECL (Thermo Fisher) and captured on an imager. Quantification was done by band intensity in ImageJ and expressed as a ratio to housekeeping (GAPDH).

#### Haematoxylin and eosin staining

Paraffin-embedded tissue sections from 3D cultures mounted on slides were deparaffinized in Xylene I and II and III for 5 min before being immersed in the filtered Harris Haematoxylin for 1 min (Sigma). Tissue was then rinsed with tap water until the water was clear. Sections were then immersed in Eosin stain for 1–2 min and then rinsed with tap water until clear. Tissue was then dehydrated in ascending alcohol solutions (50%, 70%, 80%, 95%×2, 100%×2) and cleared with xylene (2×). Tissue sections were then mounted in mowiol (sigma) and coverslipped ready for imaging.

#### Immunocytochemistry

At the indicated times for each experiment, 3D or monolayers cultures were rinsed free of medium with phosphate buffer solution (PBS) and immediately fixed in 4% paraformaldehyde (PFA) for a minimum of 30 min (60 min for 3D cultures) at 4 °C. For BrdU immunostaining, DNA was first denatured by incubating cultures in 2 M HCl at 37 °C for 30 min. After rinsing, non-specific binding sites were blocked with 5% donkey blocking serum in 0.25%Triton-X in phosphate-buffered saline (PBS) for 30 min at room temperature. Primary antibodies in PBS-0.1% Triton-X including rat anti-BrdU diluted 1:200 (Insight Biotechnology), mouse anti-human nestin diluted 1:200 (Millipore), rabbit anti-glial fibrillary acidic protein (GFAP) diluted 1:500 (DAKO), mouse anti-Class III β-tubulin diluted 1:500 (Sigma), rabbit anti-Class III β-tubulin diluted 1:500 (Millipore), goat anti-IL1-R (Santa Cruz), anti-rabbit Prox-1 diluted 1:500 (Millipore), mouse anti-NeuN diluted 1:1000 (Millipore), mouse anti-NeuroD1 diluted 1:200 (Abcam), rabbit anti-Sox2 (Abcam), rabbit anti-HMGB1 diluted 1:250 (Abcam) and/or goat anti-platelet-endothelial cell adhesion molecule-1 (PECAM-1) (Santa Cruz) were incubated with cells overnight at 4 °C. To identify microglia, cultures were stained with the green fluorescent-conjugated glycoprotein marker (Alexafluor IB4+) (2.5 μg/ml) (Invitrogen).

Primary antibodies were probed using appropriate secondary antibodies conjugated with Alexa 488, 555 or 647 (Life Technologies). Cultures were then washed once with PBS before being mounted in mowiol (Sigma-Aldrich). Optimum antibody concentrations were determined by testing each antibody on cultures, and these dilutions mentioned above for each antibody are the concentrations of antibody that provided optimal signal. For each experiment, negative controls were obtained to rule out any non-specific secondary antibody binding. For monolayer cell cultures, cells were immunostained in the same fashion with PBS 0.1% Triton-X rather than 0.25%. After completion of immunostaining, cells were counterstained with the nuclear stain 4′,6-diamidino-2-phenylindole (DAPI; 5 μg/ml) (Sigma) for 6 min ready for imaging and counting.

#### Imaging, cell counting and statistical analysis

Imaging was performed on an inverted DM IRB microscope (Leica Microsystems, Milton Keynes, UK) using Open Lab 2.1 (Improvision, Lexington, MA, USA). The area of a 20× field was measured using a 255 μm grid graticule slide (Microbrightfield, Williston, USA). Cell counting was performed on 6 random 20× fields per 3D culture disc or cover slip in monolayer cultures using the Open Lab image-capturing system version 2.1 (Improvision, Lexington, MA, USA). Raw data were averaged and plotted ± SEM and expressed as cells/mm^2^ per 3D culture or cover slip, based on a sample of four to eight per condition per repeat. GraphPad Prism was used for data analysis (GraphPad Inc. USA). (*n*) refers to the number of independent patient samples used to generate cultures. Statistical significance was assessed using Student’s *t* test for single and one- or two-way analysis of variance (ANOVA) with Bonferroni post-hoc test for multiple comparisons, at *p* < 0.05. A Leica SP5 laser scanning confocal microscope (Leica DMI600 inverted microscope frame) was used to obtain high-resolution images and confirm genuine staining using orthogonal z-stacks and sequential scanning with specific channels for Alexa-488 and Alexa-555.

## Results

### Patients’ characteristics

All patients (*n* = 15) had drug refractory complex partial seizures with hippocampal sclerosis and underwent epilepsy surgery at the Wessex Neurological Centre, Southampton, or University Hospital Wales, Cardiff, performed by WPG.

Using standardised and validated battery of neuropsychological tests (see [33] for a review), including Logical Memory I and II (LM I and LM II), Verbal Paired Associates I and II (VPA I and VPA II), the Rey Complex Figure Test (ROCF) and the Warrington Recognition Memory Test for Words (RMT-W), poor verbal and non-verbal memory impairment were observed in 8 and 7 patients, respectively (Table 1).

### Both the tissue microenvironment and endogenous precursor cells are preserved in hippocampal 3D cultures from adult human brain

Haematoxylin & eosin staining showed that within 21 DIV (days in vitro), cells re-aggregated into a three-dimensional (3D) tissue-like structure (Fig. 1A). Consistent with preservation of the cellular components of the in vivo dentate gyrus stem cell niche, adult 3D cultures have sox-2^+^, nestin^+^GFAP^+^ cells as well as Nestin^+^GFAP^-^ (type 2a) cells consistent with Type 1 radial glia-like cells and Type 2a transient amplifying cells respectively [34] (Fig. 1B). Cultures also contained cells immunostaining for astrocytes (7.3 ± 0.5%) (Fig. 1C), markers of mature neurons (19.3 ± 1.0%) (Fig. 1D), small numbers of microglial cells (Fig. 1E) in addition to many endothelial cells expressing the platelet-endothelial cell adhesion molecule-1 (PECAM) a marker (Fig. 1F). We also observed many early NeuroD1^+^ neurons consistent with Type 3 neuroblasts. Some of these cells incorporated the proliferation marker EdU, in a pulse and chase protocol, confirming in-vitro generation (Fig. 1G), and co-expressed the granule cell marker Prox1^+^ [35] (Fig. 1H). Neuronal maturation in 3D cultures was evident by the observation of NeuN^+^EdU^+^ cells after a BrdU pulse and chase protocol (Fig. 1I). Together, these observations show that human hippocampal 3D cultures contain the key cell subtypes involved in the in vivo stem cell niche and supports ongoing in-vitro neurogenesis. We therefore used this model to investigate human neurogenesis in the microenvironment of the sclerotic hippocampus.

### The 3D sclerotic hippocampal culture microenvironment is anti-neurogenic

Reduced neurogenesis in rodent models of chronic temporal lobe epilepsy (TLE) is due to a decline in the neuronal fate-choice decision of newly generated cells [36], a component of which may be epigenetically modulated [4], but which might also be driven by an altered microenvironment [36]. We began to address the latter hypothesis by comparing the neurogenic potential of matched human hippocampal precursors from the same patient (Fig. 2A), cultured either in 3D within their sclerotic tissue microenvironment (Fig. 2B) or cultured under ideal growth conditions using a standard neurosphere protocol (Fig. 2E) [37, 38].

Comparing neurogenesis in such disparate models is challenging so we used a proportional assay of proliferating cells that differentiated into neurons. To determine neurogenesis under ideal conditions (Fig. 2E–H), secondary generated neurospheres derived from single proliferating cells were dissociated, allowed to differentiate over 10 days, and the proportion of generated neurons determined. Neurogenesis by patient-matched precursor cells grown in their tissue environment was assessed in 3D cultures using an early BrdU pulse and 21-day chase paradigm and the proportion of BrdU cells staining for TuJ1 determined (Fig. 2B, C).

We found encouraging evidence of an environmental effect with only 3.6 ± 0.8% of early proliferating cells becoming neurons in 3D culture compared to 36.14 ± 7.5% of patient-matched proliferating cells generated from neurospheres culture (Fig. 2D).

### 3D hippocampal cultures recapitulate the inflammatory environment of their parent tissue and IL1-β-IL-1R axis mediates the anti-neurogenic effect of the sclerotic adult human hippocampal microenvironment

Consistent with animal studies, post-mortem studies have demonstrated that neurons, astrocytes, microglia and endothelial cells are involved in sustaining a pro-inflammatory microenvironment in chronic temporal lobe epilepsy [21]. The pro-inflammatory cytokine IL1-β is a key mediator in the pathogenesis of mesial temporal lobe epilepsy (see [39] for review), is increased in human temporal lobe epilepsy tissue [19] and inhibits hippocampal neurogenesis in mice [24]. We therefore sought to investigate the hypothesis that IL1-β-IL-1R axis is active and whether it contributes to the microenvironment anti-neurogenic effect in adult human hippocampal sclerotic 3D cultures.

**Fig. 3:**
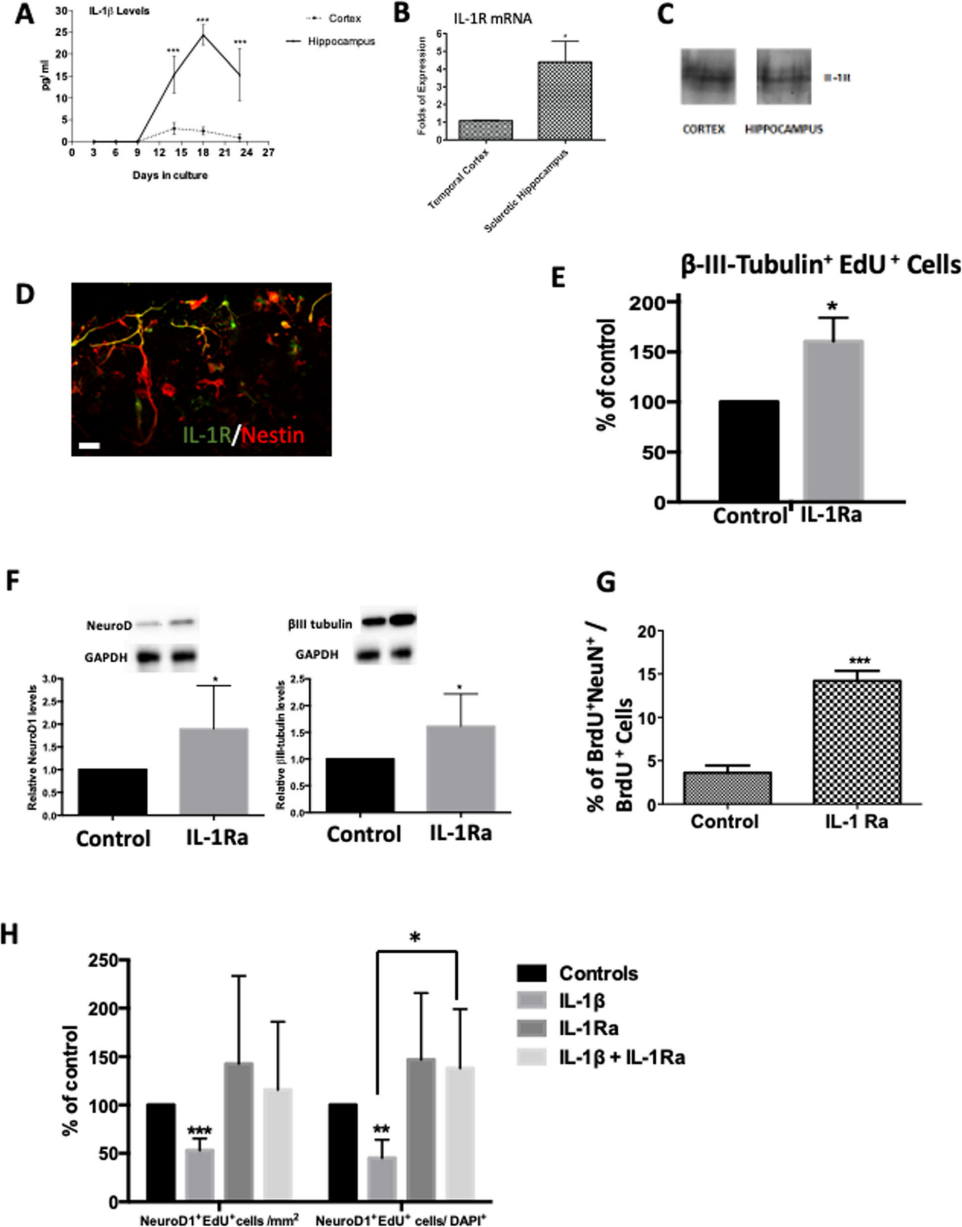
IL-1β and IL1R interact to mediate sclerotic hippocampal antineurogenic effects. **A** ELISA assay analysis in cultures’ supernatants showed that sclerotic hippocampus derived 3D cultures secrete significantly more IL1-β than cortical counterparts at the indicated time points. **B** Levels of IL1-β receptor (IL1-R) mRNA are significantly higher in hippocampal cultures. **C** IL1-R protein is expressed in cell lysates of both hippocampal and cortical 3D cultures. **D** IL1-R is widely expressed by nestin+ precursor cells in hippocampal 3D cultures. (Scale bar 50 μm). **E** Blocking endogenous IL1-β by co-treatment with IL-1Ra in 3D hippocampal cultures increases the formation of new neurons (Class III-β-tubulin+EdU+) by 63 ± 16% compared to control cultures. **F** Significant increase in neurogenesis is also evident by higher levels of the immature (early) neuron proteins NeuroD1 (89% increase) and class III-β-tubulin (74% increase) as measured by western blots. **G** The proportion of newly formed post-mitotic BrdU+NeuN+ neurons also increased following 3D hippocampal culture treatment with 100 ng/ml of IL-Ra. **H** In monolayers (2D) cell cultures, addition of exogenous IL1-β causes a significant decrease in density and proportions of new neurons (Class III β-tubulin+EdU+). Addition of the IL-1R blocker IL-1Ra completely blocks this antineurogenic effect. Values are means ± SE. Comparisons made using student’s *t* test for single comparisons (**B**, **E**, **F**, **G**) and one (**H**) or two-way ANOVA (**A**) with Bonferroni post-hoc for multiple comparisons with *p < 0.05, **p < 0.01, ***p < 0.001 considered significant. Each experiment included at least 12 3D cultures (discs) from 3 different patients

To address this question, 3D cultures were generated from sclerotic hippocampal tissue and histologically normal cortex from the same patient, to act as a technical control (showing that the inflammatory changes observed were not due to culturing methodology). Our ELISA quantification of cell culture supernatant showed that whilst IL1-β levels remained low (3.6 ± 1.43 pg/ml at 14 DIV) in cortical 3D cultures, it significantly increased in hippocampal 3D cultures peaking at 24.4 ± 2.3 pg/ml by day 18 before starting to drop to 15.3 ± 5.9 pg/ml by 23DIV (*n* = 3) (*p* < 0.05, two-way ANOVA) (Fig. 3A). This is consistent with the previous demonstration of raised IL1-β levels in the hippocampus of TLE patients with hippocampal sclerosis [19] and shows that the culturing methodology itself did not generate significant inflammation.

Quantitative PCR of its canonical receptor IL-1R demonstrated a more than 4-fold increase in sclerotic hippocampal 3D cultures compared to their cortical counterparts (Student’s *t* test, *p* < 0.05) (Fig. 3B), with Western blot confirming tissue expression in both cortical and hippocampal cultures (Fig. 3C), and confocal immunostaining showing dense IL1-R expression by nestin-expressing progenitor cells in sclerotic hippocampal 3D cultures (Fig. 3D).

To examine the involvement of IL-1R in mediating reduced neurogenesis in the hippocampal 3D cultures, endogenous IL-1β activity was blocked using the competitive 100 ng/ml IL-1R antagonist IL-1RA, resulting in a significant increase in the number of newly-formed neurons (Class III β-tubulin+EdU+) (density: 163 ± 16% of control, *p* = 0.02, proportion: 142 ± 15% of control, *p* = 0.05; Student’s *t* test) (Fig. 3E) (*n* = 3). These results were also confirmed by western blotting for neuronal proteins, with both NeuroD1 at 8 DIV and Class III β-tubulin at 18DIV showing 89% and 74% more protein, respectively, following treatment with IL-1RA (*p* < 0.05, Student’s *t* test) (Fig. 3F).

As TuJ1 is an early marker of neurogenesis, 3D sclerotic hippocampal cultures were also processed for the mature post-mitotic neuronal marker NeuN co-expression. Neurogenic rescue was again confirmed by significantly increased numbers of (BrdU+NeuN+ cells) in hippocampal 3D cultures treated with 100 ng/ml of IL1-R1 antagonist IL-1Ra (4.8 ± 0.7% under control conditions vs. 14.23 ± 1.13 in cultures treated with IL-1Ra, *p* < 0.05, Student’s *t* test) (Fig. 3G).

To confirm the effect of IL-1β on reducing hippocampal neurogenesis, cells isolated from sclerotic hippocampus were grown in monolayer (2D) cultures. Addition of 10 ng/ml of IL-1β at 2 h post platting for the duration of experiment significantly decreased the numbers (to 58 ± 4% of control, *p* < 0.001) and proportion (to 51 ± 16% of control, *p* = 0.003) of newly born (NeuroD1+EdU+) neurons at 6DIV (Fig. 3H). Treatment with IL-1Ra (100 ng/ml) alone did not significantly increase neurogenesis (implying a lack of endogenous IL1R1 activation in monolayer cultures); however, when co-applied at 2 h of platting, it completely abolished the IL-1β-mediated anti-neurogenic effect on the proportions of NeuroD1+EdU+/ EdU+ cells (density: 85 ± 15% of control, *p* = 0.05, proportion: 114 ± 16% of control, *p* < 0.01) (Fig. 3H); confirming IL-1R signalling of the IL-1β anti-neurogenic effect (*p* < 0.05, one-way ANOVA with Bonferroni post-hoc test).

Together, these results demonstrate that IL1-β is actively secreted in primary 3D cultures of epileptic human hippocampus, is a powerful mediator of reduced hippocampal neurogenesis via the IL1-R1 receptor, and that this altered neurogenesis can be pharmacologically reversed using IL1Ra in vitro*.*

### Secretion of HMGB1 modulates the anti-neurogenic of IL-1β

In animal models of mTLE, there is substantial evidence supporting crosstalk between IL-1β-IL1R1 and HMGB1/TLR pathways before they converge at a common MyD88 pathway to enhance inflammation via nuclear factor kappa-B (NFκB) activation [21, 40]. Increased HMGB1 expression during a seizure activity has been suggested to support the secretion of a second and sustained wave of extracellular HMGB1; both waves may enforce an autocrine and paracrine loop leading to hyperexcitability and sustain inflammation via IL-1β [21].

**Fig. 4:**
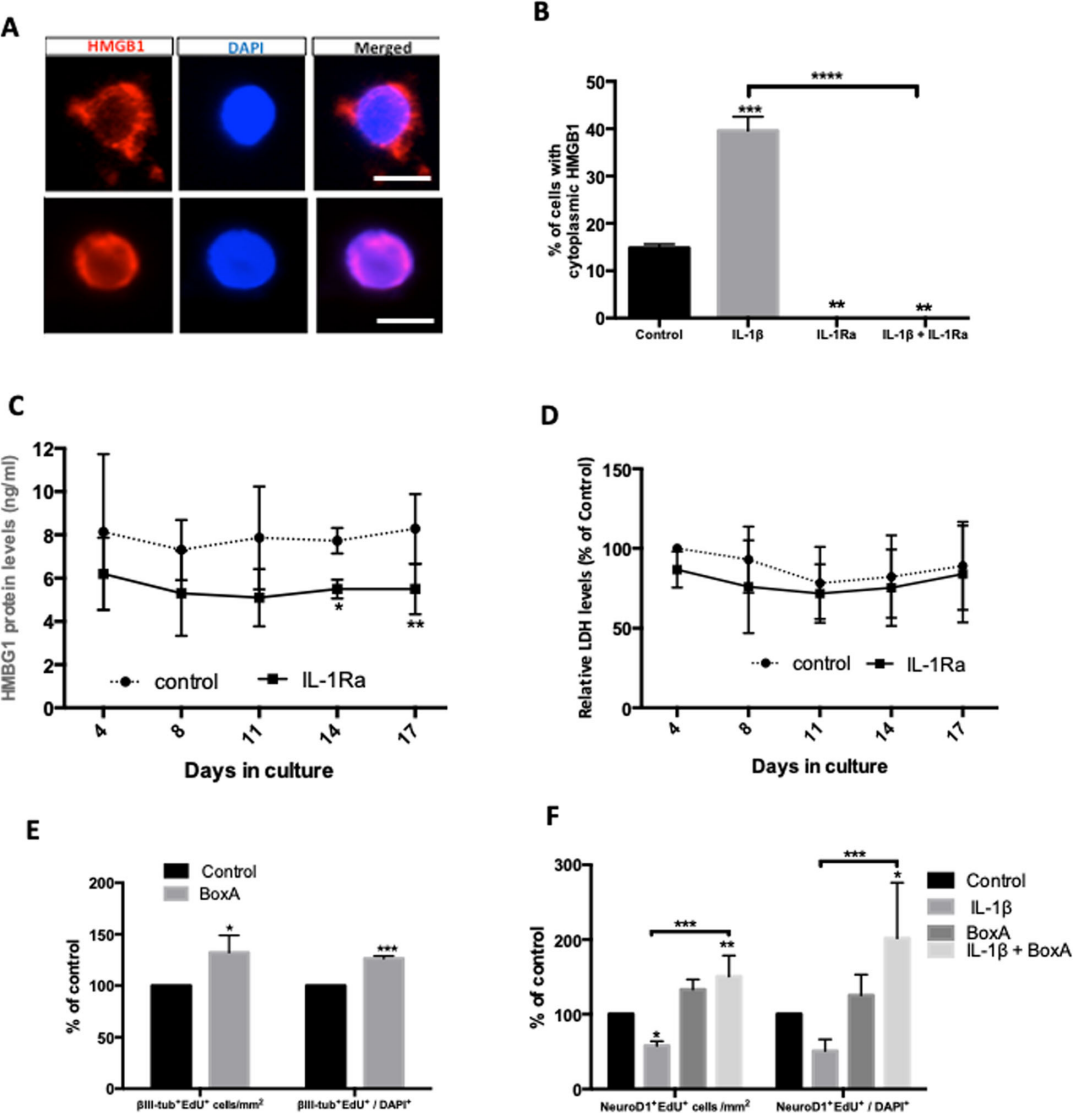
HMGB1-IL1-β-IL1R mediates the anti-neurogenic effect of the sclerotic hippocampal microenvironment. **A** Representative images of immunocytochemistry for cellular localization of HMGB1 (red) and DAPI (blue) in 2D hippocampal monolayer cultures. Top panel depicts cytoplasmic pattern of HMGB1 staining whilst the bottom one shows a nuclear localisation of HMGB1 (Scale bar 10 μm). **B** Treatment with 10 ng/ml IL-1β significantly induced a cytoplasmic phenotype, which can be blocked with IL-1Ra. **C** Secretion of HMGB1 from 3D hippocampal cell cultures as measured by ELISA significantly decreased with IL-1Ra treatment at 14 and 17 DIV. **D** No difference in cell death between controls and IL-1Ra-treated 3D cell cultures as measured by LDH assay for the duration of the experiment. **E** Blocking HMGB1 signalling in 3D hippocampal cultures increased neuronal cell density and proportion by 32 ± 10% (*p* < 0.05) and 26 ± 1% *p* < 0.0001 respectively (*n* = 3). In 2D hippocampal monolayers, addition of Box-A completely blocked the anti-neurogenic effect of IL-1β. Values are means ± SE. Comparisons made using student’s *t* test for single comparisons (**E**) and one (**B**, **F**) or two-way ANOVA (**C**, **D**)with Bonferroni post-hoc for multiple comparisons with *p < 0.05, **p < 0.01, ***p < 0.001, ****p < 0.0001, considered significant. Each experiment included at least 12 3D cultures (discs) from 3 different patients

We therefore explored whether the IL-1β anti-neurogenic effect is mediated through interaction with HMGB1. To address this, we treated primary hippocampal 2D cultures with IL-1β and/or IL1Ra for 18 h before determining the cellular localisation of HMGB1 (Fig. 4A). Under control conditions, there was a significant proportion of cells with HMGB1 predominantly localized to the nucleus (Fig. 4B). IL-1β treatment resulted in significant nuclear to cytoplasmic translocation; while co-treatment with IL1Ra completely prevented this (*p* < 0.05, one-way ANOVA with Bonferroni post-hoc) (Fig. 4B). Given the possibility of consequent cellular secretion, we assayed HMGB1 levels from 3D hippocampal culture medium, observing a steady release which was again reduced after treatment with IL-1Ra (Fig. 4C) (*p* < 0.05, two-way ANOVA), and which was not associated with reduced cell death (*p* > 0.05, two-way ANOVA) (Fig. 4D), suggesting active secretion by live cells rather than passive HMGB1 release from necrotic cells [41].

We then examined HMGB1 effects on neurogenesis using its antagonist Box-A [21], again in monolayer cultures. Box-A treatment significantly increased both cell counts and proportions of newly born class-III-β tubulin+/EdU+ cells at 18 DIV (*p* < 0.05, Student’s *t* test) (Fig. 4E). To explore possible crosstalk between IL-1β and HMGB1, hippocampal cells were treated with IL-1β in the presence or absence Box-A. Interestingly, the blockade of HMGB1 signalling with Box-A completely reversed the anti-neurogenic effects of IL-1β, with significant increase (50%) in total cell counts of NeuroD1+/EdU+ cells compared to control at 6 DIV (Fig. 4F, *p* < 0.05, one-way ANOVA with Bonferroni post-hoc) suggesting the necessity of active HMGB1 signalling for the antineurogenic effect of IL-1β.

## Discussion

We have previously shown that restoring neurogenesis using the antidepressant Fluoxetine to directly drive stem cell proliferation, completely corrects spatial learning impairment [14], suggesting that abnormal neurogenesis is a key mechanism, but shedding little light on the underlying signalling processes to allow a more rational targeted pharmacological approach.

Here, we utilise novel 3D cell cultures grown from surgical samples resected from patients with memory impairment and hippocampal sclerosis causing mTLE, to demonstrate that endogenous neurogenesis is reduced by the parent tissue-inflamed-microenvironment. This effect is at least partly mediated via the IL-1β-HMGB1-IL1R signalling pathways, thus identifying the IL1-R, TLR4 and receptor for advanced glycation end-products (RAGE) receptors and their pathways as valid pharmaceutical targets. We further show a complex relationship between IL-1β and the danger signal/master inflammatory regulator HMGB1, whereby IL-1β regulates HMGB1 intracellular translocation and release and HMGB1 appears necessary for the overall antineurogenic effect of IL-1β.

### 3D cultures maintain both a functioning neurogenic niche and the inflammatory environment of mTLE

Our 3D cultures contained Sox-2 expressing hippocampal precursor cells (Fig. 1B) and demonstrated active in-vitro granule cell neurogenesis with EdU+ Prox-1+ cells specific to this lineage [35] surviving for over 3 weeks in culture. This functional neurogenic model was further validated by the presence of the key supporting cellular components of the in-vivo stem cell niche (Fig. 1C–I), all of which regulate cell fate decision under physiological conditions [42].

Neuroinflammation primarily driven by astrocytic and microglial activation has long been implicated in epileptogenesis and hippocampal neuronal loss [21, 39] with IL-1β and its type 1 receptor (IL-1R) broadly expressed by astrocytes, microglia and neurons [24]. Our hippocampal 3D culture paradigm clearly recapitulated this innate inflammatory environment. Histologically, it had the requisite cellular machinery of neurons, astrocytes and microglia, all of which produce inflammatory cytokines in mTLE [19, 21]. Functionally, it showed evidence of sustained activation with constitutive IL-1β and HMGB1 release (Fig. 3A and Fig. 4C), and upregulation of IL1-R receptor mRNA (Fig. 3B) compared to normal cortical tissue from the same patient. We recognise this as a limitation; however, normal cortex is the best available control we can obtain in the setting of human brain tissue, from the perspective of a non-inflamed environment. Importantly, this innate immune activation was not an artifact of culture generation as there was significantly lower constitutive IL-1β release from 3D cultures of patient-matched histologically normal cortex (Fig. 3A), although a component of this cannot be completely ruled out. The validity of 3D culture is further supported by the contrasting lack of any effect of IL-1Ra in patient matched 2D vs. 3D hippocampal cultures (Fig. 3E and F). Moreover, our results extend previous observations of focal increases in IL-1β immunostaining in human mTLE tissue [19, 43] by also showing direct evidence of IL-1β-mediated increase in HMGB1 nuclear to cytoplasmic intracellular translocation and extracellular release.

In summary, our 3D cultures maintained both constitutive granule cell neurogenesis and significant innate immune system activation, making it suitable for studying the effects of the latter on the former.

### Neurogenesis is impaired by endogenous constitutive IL-1β release

Our findings of an anti-neurogenic effect in human tissue are consistent with that in animal models where IL-1R receptors are highly concentrated in the neurogenic niche of the dentate granular cell layer [24], IL-1β is potently anti-neurogenic [24] and high levels of exogenously administered IL-1β inhibits memory formation [44]. Treatment of hippocampal 3D cultures with the IL1R antagonist IL1-Ra resulted in a doubling of the percentage of newly born neurons (Class III- β tubulin+EdU+) and (NeuN+BrdU+) (Fig. 3E), clearly implicating IL-1β as an antineurogenic signal, a hypothesis confirmed by direct application in monolayer dissociated cultures and again reversal by IL-1Ra. Interestingly, there was no effect of IL1-Ra under dissociated culture control conditions implying a lack of constitutive IL-1β release—implying either a cell density effect and/or the necessity for cell-cell communication for the maintenance of the inflammatory state (*vide infra*). From a pharmacological perspective, and in contrast to previous literature suggesting that high concentrations of IL-1Ra (up to 100-fold) in excess of IL-1β are required to inhibit its biological effects in mice monocyte cell cultures [45], we observed that the antineurogenic effects of IL-1β were completely abolished at 100 ng/ml (10-fold of IL-1 β) in our 2D primary human cortical cell cultures (Fig. 3H). Our finding of a lower effective inhibitory dose of IL-1Ra is pharmacologically reassuring and may simply be a reflection of cell system or species specific effects.

### Constitutive HMGB1 release is antineurogenic in 3D culture and is necessary for the IL-1β antineurogenic effect in dissociated culture

High mobility group box 1 (HMGB1) is a highly conserved nuclear protein found in all cell types, and is one of the damage-associated molecular pattern molecules (DAMPs) that allows the sensing of cell stress [46]. Physiologically, it is a nuclear located chromatin-binding protein either actively released under cell stress or passively released after cell death/lysis, triggering inflammation [41]. Released HMGB1 undergoes redox changes to activate the TLR4 complex signalled via the MyD88 pathway to nuclear NF-kB activation, which is also activated by IL-1β-IL1-R signalling [22]. Extracellularly, it can also bind to other proinflammatory molecules such as DNA, RNA and IL-1β [46, 47], then binding to the receptor for advanced glycation end-products (RAGE) to undergo endocytosis with its bound partner-molecule complexes to activate intracellular cognate receptors mediating inflammasome assembly and cytokine release [48, 49]. RAGE is a pro-inflammatory type I transmembrane receptor and a member of the immunoglobulin gene superfamily, present in many cell types mainly in a preformed intracellular pool that can be rapidly transported to the cell surface during cell activation [50]. This RAGE receptor-mediated internalisation is specifically blocked by the HMGB1 antagonist Box-A [51]. Thus, HMGB1 acts bi-directionally to alert the environment about cell stress and cells about environmental stress.

We observed a persistent release of HMGB1 in 3D culture medium over a period of 17 DIV, which was not due to cell death (Fig. 4D) and exposure of our 3D hippocampal cultures to the HMGB1 antagonist Box-A increased neurogenesis (Fig. 4E), raising the possibility of a HMGB1 mediated anti-neurogenic effect. The cellular origin of HMGB1 in 3D sclerotic cultures is likely to be astrocytes and microglia given their increased levels in sclerotic human hippocampal tissue.

Interestingly, we found that IL-1β increases HMGB1 translocation from the nucleus into the cytoplasm in dissociated human hippocampal mixed-cell cultures, a phenomenon previously reported to be signalled by interferon [52] and believed to be a critical step in active cellular HMGB1 release in response to cell stress. Given the constitutive release of both IL-1β and HMGB1 in 3D cultures, we next examined the effect of IL-1Ra on HMGB1 release and found that it was reduced (Fig. 4C) whilst having no effect on cell death (Fig. 4D), implying an effect on active cellular release. This raised the possibility that the antineurogenic effects of IL-1β could at least be partly mediated via HMGB1.

Interestingly, the HMGB1 antagonist Box-A completely abolished the anti-neurogenic effect of IL-1β in dissociated culture, suggesting a complex bi-directional interaction between IL-1β and HMGB1 signalling on neurogenesis. If IL-1β acted purely through IL1-R activation in monolayer cultures, then Box-A would not be expected to reverse its antineurogenic effect. However, it has been recently shown that Box-A specifically blocks HMGB1-RAGE binding and uptake [51] without affecting HMGB1/TLR4 inflammation mediated via the dimerisation and activation of TLR4 adapter protein MD-2 [53]. Since RAGE binds both IL-1β and HMBG1 before cellular uptake to trigger an amplified inflammasome release, this potentially explains the complete reversal of the IL-1β antineurogenic effect by Box-A in dissociated cultures. In this 2D paradigm, where constitutive IL-1β release is negligible (Fig. 3H), exogenous IL-1β likely acts predominantly by causing HMGB1 release which then binds to IL-1β, undergoes a largely autocrine reuptake with amplification of further cytokine and HMGB1 release and eventual antineurogenic signalling on IL1-R-positive progenitors via IL1-R1 and TLR4 [51]. In our 3D cultures, a parallel direct effect of HMGB1 activation of TLR4 receptors [53, 54] cannot be ruled out, and these experiments will form the basis of future work requiring specific blockers of HMGB1/TLR4 signalling.

In our 3D cultures, paracrine effects may be more active due to the 3D cellular structure and again it is interesting to note the increasing IL1-β release over time, suggesting some autocrine/paracrine amplification of innate immune system activation. Given the substantial increase in cytoplasmic HMGB1 staining in GFAP-positive astrocytes observed in hippocampal surgical specimens of patients with mTLE with hippocampal sclerosis [21], astrocytes are likely to play a significant role in these proposed paracrine effects as a primary source of HMGB1 release. A significant contribution of neurons and microglia to these effects cannot be excluded either. It is also relevant to note that the rapid translocation of RAGE to the cell surface during inflammatory activation is partly under NF-kB control [55, 56] which is signalled via both the TLR4 and IL-1β-IL-1R pathways to MyD88, adding a further amplification pathway to that of RAGE-mediated uptake with further inflammasome release and pyroptosis.

The redox state of released HMGB1 is critical for its pro-inflammatory actions, and although we were unable to measure the redox state of HMGB1 in our culture medium, likely due to its binding to partner-molecule complexes [47], it is highly likely that our 3D cultures contained extracellular di-sulphide HMGB1 as this is the isoform necessary for TLR4 [53] and involved in RAGE activation [51].

Taken together, these observations demonstrate that HMGB1 and IL1-β are key mediators of the anti-neurogenic effect of the inflammatory microenvironment in the adult human hippocampus in patients with hippocampal sclerosis and mesial TLE.

## Conclusions and clinical implications

Medial temporal lobe epilepsy is a common and often intractable form of epilepsy that is associated with long-term morbidity in the form of an increased incidence of depression and problems with learning and memory that may progress despite adequate seizure control [57]. We now provide evidence in primary human 3D cultures of constitutive antineurogenic signalling via the IL1-β-IL-1R and HMGB1 RAGE/TLR4 pathways and demonstrate pharmacological reversibility in tissue from patients with severe long-standing drug refectory epilepsy and memory impairments. Whilst blockade of these pathways has been shown to improve seizure control in animal models, our results raise the possibility that currently licensed (IL1-Ra) and/or future developed HMGB1 pathways inflammatory blockers blocking HMGB1/RAGE interactions (Box-A) or TLR4 mediated inflammation (P5779) [51] may be able to restore hippocampal neurogenesis and improve aspects of hippocampal learning in patients with established mTLE.

## Data Availability

All the datasets and materials supporting the conclusions of this article are included within the article.

## Abbreviations

ANOVA: Analysis of variance
BrdU: 5-Bromo-2′-deoxyuridine
cDNA: Complementary deoxyribonucleic acid
DAPI: 4′,6-Diamidino-2-phenylindole
DIV: Days in vitro
2D: Two-dimensional
3D: Three dimensional
DMEM: Dulbecco’s modified Eagle medium
EdU: 5-Ethynyl-2′-deoxyuridine
ELISA: Enzyme-linked immunosorbent assay
GFAP: Glial fibrillary acidic protein
HMGB1: High mobility group box 1
IL-1R: Interleukin-1 receptor
IL-1β: Interleukin-1 beta
IL1Ra: Interleukin-1 receptor antagonist
mTLE: Mesial temporal lobe epilepsy
NFκB: Nuclear factor kappa-B
PCR: Polymerase chain reaction
RAGE: Receptor for advanced glycation end-products
TLE: Temporal lobe epilepsy
TLR 2/4: Toll receptors 2/4
TuJ1: Class III β-tubulin
